# Regorafenib-induced renal-limited thrombotic microangiopathy: a case report and review of literatures

**DOI:** 10.1186/s12882-021-02656-9

**Published:** 2022-03-19

**Authors:** Qinghua Yin, Na Guo, Xueli Zhou, Huan Xu, Song Lei, Ping Fu, Hui Zhong

**Affiliations:** 1 Kidney Research Institute, Division of Nephrology, West China Hospital, Sichuan University, Chengdu, 610041 China; 2 Department of Pathology, West China Hospital, Sichuan University, Chengdu, 610041 China

**Keywords:** Nephrotic syndrome, Regorafenib, Thrombotic microangiopathy (TMA), Tyrosine kinase inhibitors (TKI), Case report

## Abstract

**Background:**

Regorafenib belongs to a sub-group of small-molecule multi-targeted tyrosine kinase inhibitors(TKIs). In various studies with respect to the side-effect of regorafenib, drug-associated proteinuria standardly qualified to be defined as nephrotic syndrome was rarely reported as well as the relation of regorafenib with the occurrence and development of thrombotic microangiopathy (TMA).

**Case presentation:**

In this case report and literature review, we presented a 62-year-old patient receiving regorafenib for metastatic colon cancer, manifesting abundant proteinuria, in which TMA was also diagnosed through renal biopsy. As far as we were concerned, this was the first reported in terms of regorafenib-induced TMA confirmed by renal biopsy.

**Conclusion:**

This case indicates that regorafenib, a kind of TKIs may result in TMA, which is a rare but life-threatening complication of cancer treatment drug. Insights from this case might help physicians diagnose rare forms of TMA and adjust treatment for patients in a timely manner.

## Background

Regorafenib is an orally-taken small-molecule multi-targeted tyrosine kinase inhibitors(TKIs)that block the activities of various protein kinases which are supposed to activate vascular endothelial growth factor receptors 1-3(VEGFR 1-3), platelet-derived growth factor receptors and fibroblast growth factor receptors by targeting tyrosine receptor kinases, and thus influence cellular metabolism, proliferation and distant migration [1]. Because of its anti-tumoral function, regorafenib has been applied around the world in metastatic colorectal cancer (mCRC). However, since proteinuria and hypertension are common side effects among all drug therapies targeting VEGF signaling and its downstream pathways, major adverse effects (AEs) should be considered with the use of such drugs [2]. In the past decade, reports have suggested that TKIs might exert multiple effects on patients’ kidney, inducing hypertension, proteinuria, acute kidney injury (AKI), and thrombotic microangiopathy (TMA) [3–6]. Among patients receiving regorafenib, hypertension occurs in 32.4% of the population, while all-grade and high-grade proteinuria are found in 7.0 and 1.4% of them respectively [7, 8]. A previous phase 3 clinical trial showed regorafenib-induced proteinuria was more frequent in Japanese individuals compared with other populations, but was generally manageable [9]. Other studies assessing regorafenib-associated proteinuria found out proteinuria rarely reached the diagnostic standard of nephrotic syndrome, and it remained unknown whether regorafenib was associated with the development of TMA. Meanwhile, studies investigating the associations between TKIs and nephrotic syndrome were scarce.

In order to supplement research on regorafenib use and morbidity of TMA, we presented a 62-year-old female receiving regorafenib for metastatic colon cancer, who developed abundant proteinuria caused by TMA (determined pathologically).

## Case presentation

**Fig. 1:**
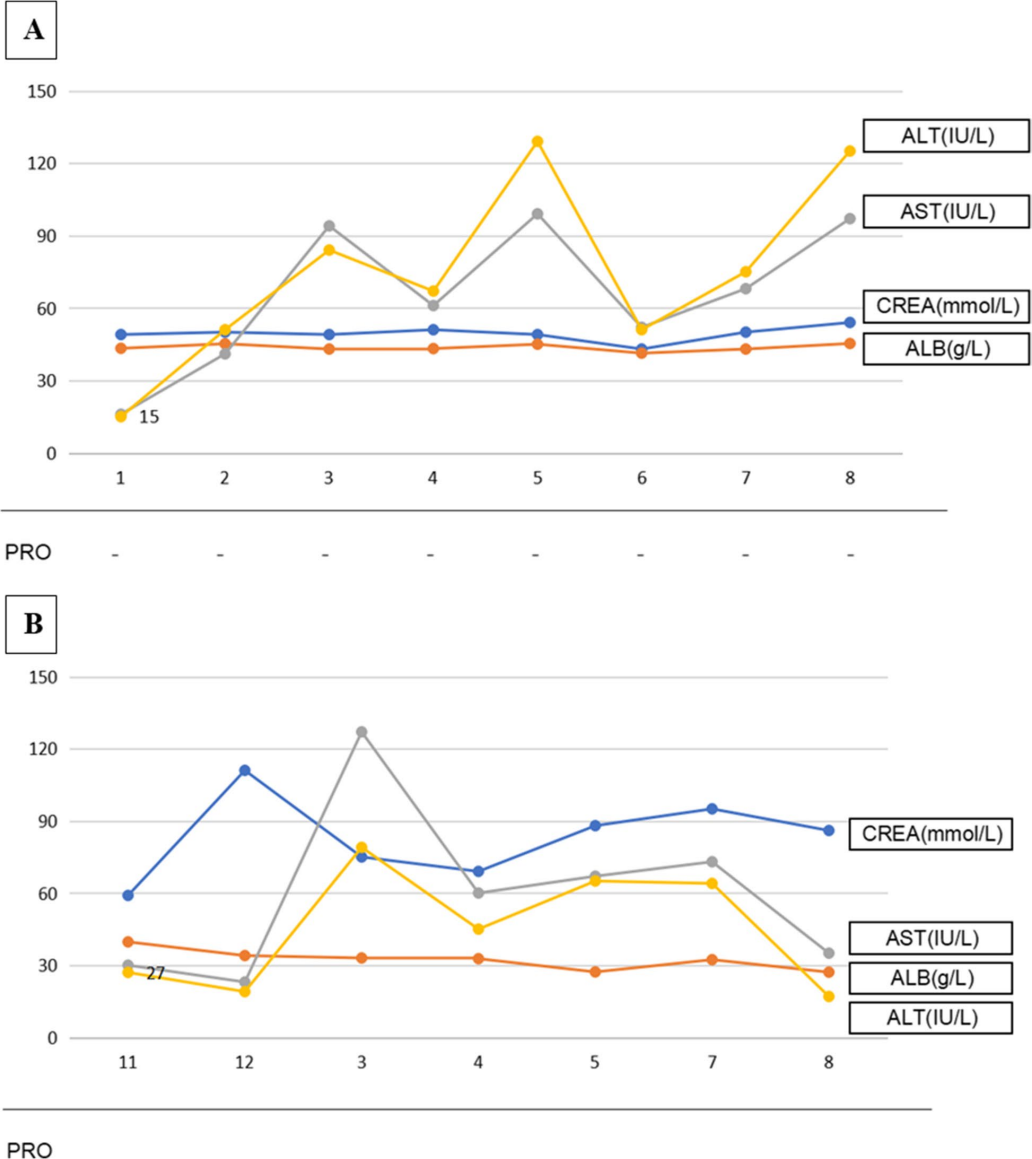
The clinical course of the patient. **A** Bevacizumab administration during an 8-month period (Jan 2017-Aug 2017). **B** Regorafenib administration during a 7-month period (Nov 2018-Dec 2018, Mar 2019-Aug 2019)

**Fig. 2:**
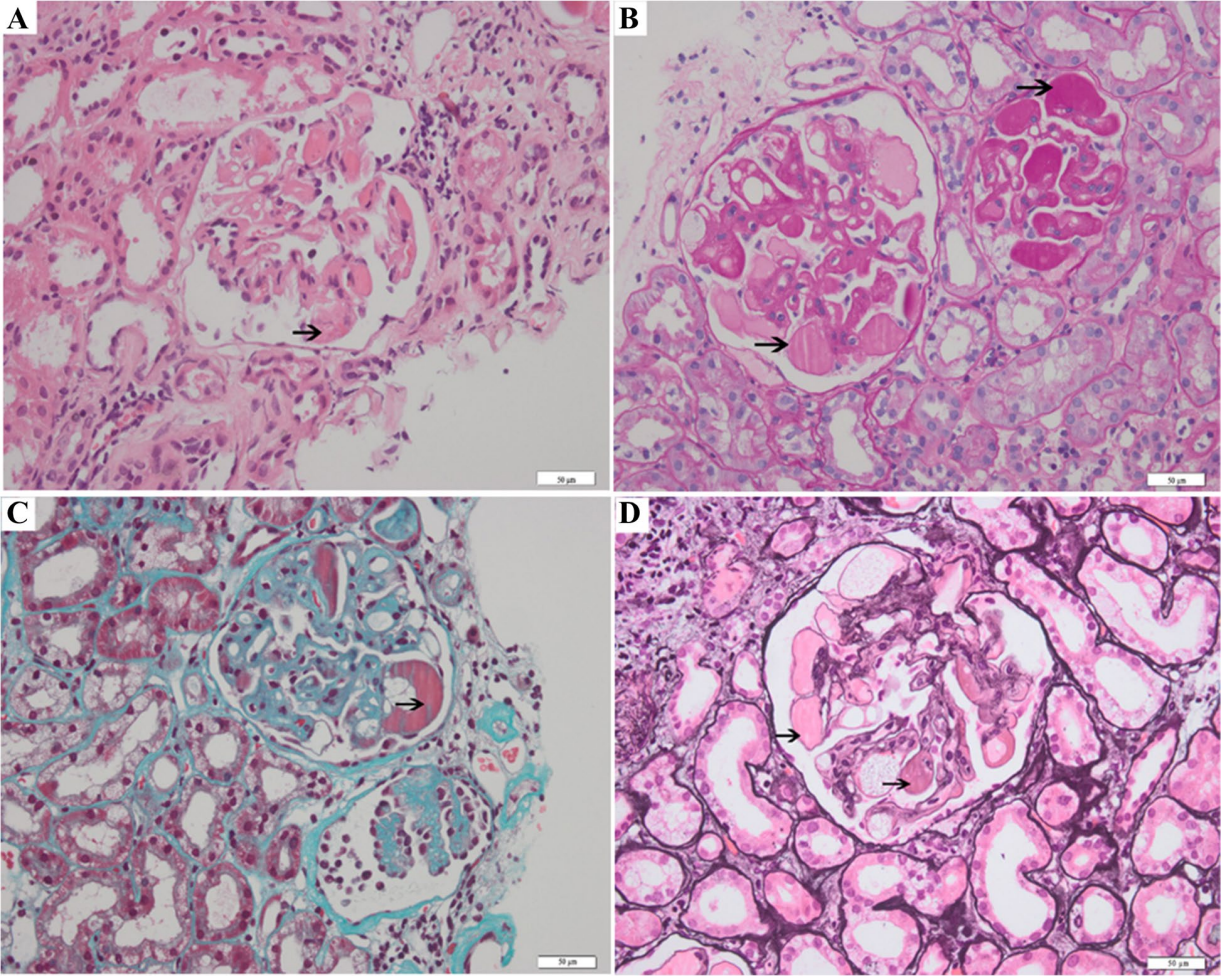
Pathological biopsy of glomerulus showing thrombotic microangiopathy. **A** Hematoxylin-eosin staining×200. **B** Periodic Acid–Schiff staining×200. **C** Masson’s Trichrom staining×200. **D** Periodic acid silver methenamine stain ×200. Scale bars=50μm

**Fig. 3:**
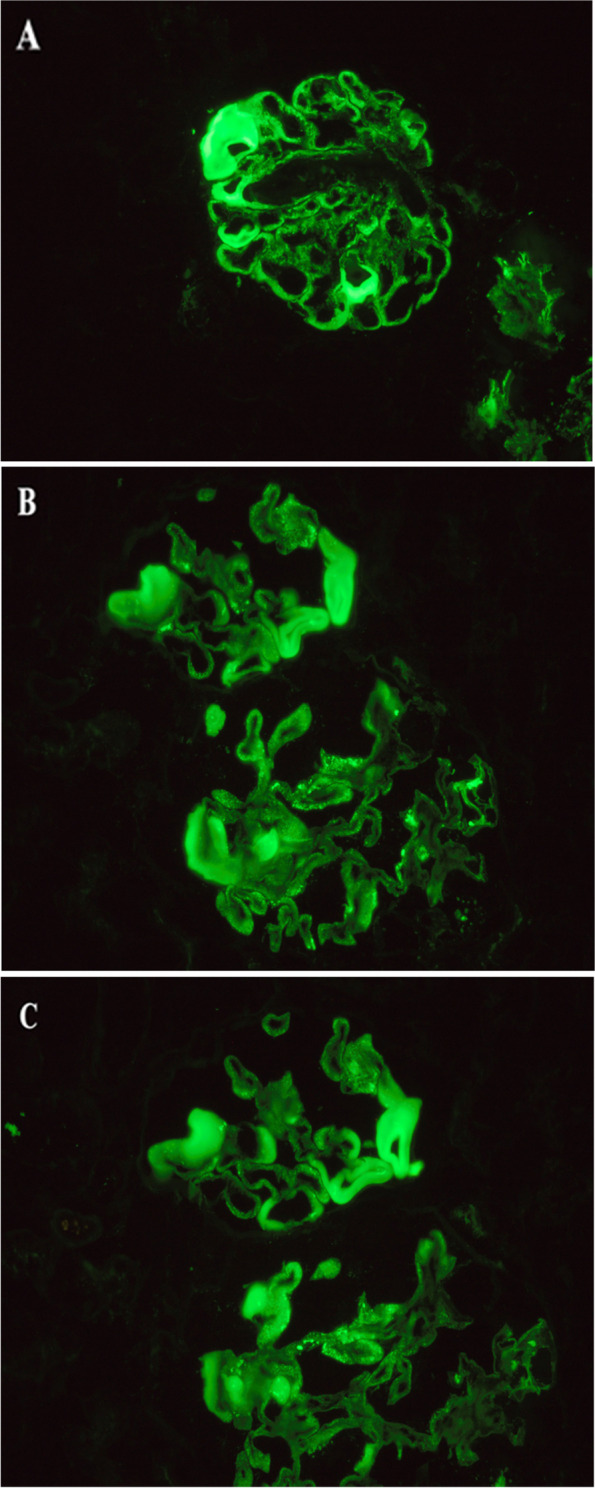
Immunofluorescence. Immunofluorescence for immunoglobulin M (**A**), kappa (**B**) and lambda (**C**) deposition in glomerular mesangium and glomerular capillary loops

**Fig. 4:**
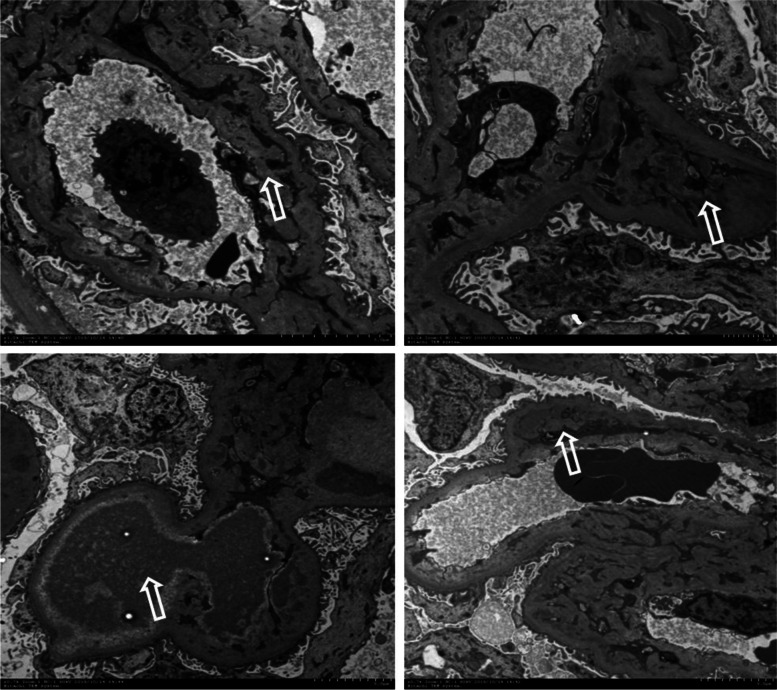
Electron micrograph. Electron microscopy revealed diffuse endothelial thickening with obliterated capillary lumina and subendothelial fluffy materials or fibrin tactoids deposition

A 62-year-old woman was diagnosed with stage IV colon cancer at hepatic flexure and lung metastasis according to the 7th edition of the American Joint Committee on Cancer (AJCC) tumor node metastasis (TNM) staging system two years ago (May 4^th^, 2016), and have been treated with combined neoadjuvant chemotherapy (imatinib, folinic acid and fluorouracil) as the primary drug regimen. Three weeks later (May 25^th^, 2016), she was admitted to the Department of Gastrointestinal Surgery for relieving her symptoms, during which tumor removal with palliative right hemi-colectomy was performed. Afterwards, FOLFIRI (5-fluorouracil, leucovorin, irinotecan) regimen which included leucovorin at 500 mg d1, fluorouracil at 500 mg d1, fluorouracil at 1700 mg civ/23h d1-2 Q2W, and irinotecan at 260 mg d1, was administered for 6 courses (July 14^th^, 2016 - Nov 24^th^, 2016). However, she still manifested progressive cancer, lung metastasis was determined as right lung biopsy showed moderately differentiated intestinal adenocarcinoma with RAS (rat sarcoma virus) genetic mutations. Therefore, bevacizumab (anti-VEGF-A antibody) (400 d1 q3w) plus 8 courses of XELOX which included oxaliplatin at 150 mg d1 and capecitabine at 1500 mg bid d1-d14 (Jan 5^th^, 2017 - Sep 25^th^, 2017) was initiated after the abovementioned treatment. Radiotherapy for pulmonary metastasis was also carried out 28 times in 2 months (Jan 8^th^, 2017 - Sep 22^nd^, 2017). Nevertheless, the tumor was not able to be contained either and continued to progress. Thus, regorafenib(80 mg qd d1-d21) was added on Jan 11^th^, 2018.In the beginning, she had normal renal function (serum creatinine, 45 μmol/L) without proteinuria or hypertension. Bevacizumab administration was performed for 8 courses spanning 8 months with transient liver dysfunction (Fig. 1A). After discontinuing bevacizumab, liver function returned to normal. However, serum creatinine gradually increased after regorafenib treatment initiation, her blood test showed hypercholesterolemia (7.11 mmol/L) and hypertriglyceridemia (2.04 mmol/L) (Fig. 1B). Moreover, she progressively developed nephrotic syndrome (serum creatinine, 91 μmol/L) in recent three months, and was thus transferred to the Department of Nephrology. On admission, her 24-hour urinary protein was 4.6 g/24h, and serum albumin was 29.1 g/L (Table 1). Regarding antibodies related to membranous nephropathy, anti-phospholipase A2 (PLA2) antibody was negative. While anti-thrombospondin type-1domain-containing 7A (THSD7A) antibody was positive. Coombs test, haptoglobin and also ADAMTS-13 activity were negative. Kidney biopsy showed fibrin thrombi in glomerular capillaries, endothelial injury with thickening, and mesangiolysis (Fig. 2A, B, C, D) revealing TMA. Meanwhile, Immunofluorescence showed IgM, kappa and lambda deposition in glomerular mesangium and glomerular capillary loops (Fig. 3A, B, C) with weak deposition of C4 and C1q. Electron microscopy revealed diffuse endothelial thickening with obliterated capillary lumina and ubendothelial fluffy materials or fibrin tactoids deposition (Fig. 4). Pathological examination indicated a diagnosis of renal-limited TMA caused by anti-VEGF drugs. After careful consultation with oncologists, regorafenib therapy was suspended. Two years following discharge from our hospital, the patient received irbesartan treatment and responded with gradual improvement of her clinical manifestation, with urinary protein 2+ and serum albumin of 33.9 g/L (Table 1).

**Table 1:** Results of laboratory test Upon admission, (May 26^th^, 2016), on admission (Oct 9^th^, 2019) and the latest follow-up (May 27^th^, 2021)

Component	Jan 16^th^, 2018 (Upon admission)	Oct 9^th^, 2019 (On admission)	May 27^th^, 2021 (Latest follow-up)
**Urinalysis results**			
Urinary protein	negative	4+	2+
24-HTP	ND	4.6	ND
**Peripheral blood**			
HB (g/L)	143	138	98
WBC (×10^9^/L)	6.38	11.78	3.43
PLT (×10^9^/L)	169	126	65
**Chemistries**			
CREA (μmol/L)	45	91	106
BUN (mmol/L)	4.6	8.3	8.6
eGFR (ml/min/1.73 m^2^)	104.6	58.7	48.3
Bilirubin (μmol/L)	14.7	7.6	6.2
LDH (IU/L)	197	249	298
TP (g/L)	74.5	51.7	56.9
ALB (g/L)	47.7	29.1	33.9
TG (mmol/L)	ND	3.19	2.25
CHOL (mmol/L)	ND	9.09	6.29
**Other**			
CRP (mg/L)	ND	11.5	ND
PCT (ng/ml)	ND	0.16	ND
Coombs Test	ND	negative	ND
ADAMTS-13	ND	negative	ND
THSD7A	ND	negative	ND

*Note*: *24hHTP* 24-hour urine protein, *HB* Hemoglobin, *WBC* White blood cell, *PLT* Platelets, *CREA* Creatinine, *BUN* Blood urea nitrogen, *LDH* Lactic dehydrogenase, *TP* Total protein, *ALB* Albumin, *TG* Triglyceride, *CHOL* Cholesterol, *eGFR* Estimate glomerular filtration rate, *CRP* C-reactive protein, *PCT* Procalcitonin, *Coombs Test* A test to detect non-agglutinating antibodies against erythrocytes by use of anti-antibodies (the Coombs’ reagent), *ADAMTS-13* A disintegrin and metalloproteinase with a thrombospondin type 1 motif, member 13, *THSD7A* Thrombospondin type 1 domain–containing 7A, *ND* No data

## Discussion and conclusion

The patient’s other causes of nephrotic syndrome including membranous nephropathy and minimal change nephropathy were ruled out according to the results of renal biopsy. Because of the patients’ renal injury appeared aftefr regorafenib treatment, combing with the results of renal biopsy, we also exclude a paraneoplastic nephrotic syndrome. The current case indicated that kidney damage associated with regorafenib coule lead to nephrotic-range proteinuria and histopathological TMA limited to the kidney unlike systemic TMA, which was associated with thrombotic thrombocytopenic purpura [10]. Most recently, kidney-related adverse event of regorafenib had been reported as lupus-like glomerulonephritis, which presented with acute kidney injury, proteinuria, and hematuria [6]. However, nephrotic syndrome with or without TMA characteristics has seldom been reported as a severe side effect of TKIs in adults with cancer.

It was widely acknowledged that solid tumors were characterized by rapid growth and abundant blood supply. Therefore, it was a recommended therapeutic to inhibit vascular development in order to treat solid tumors. The main targets of anti-angiogenesis were VEGFs, VEGF receptors (VEGFRs) and molecules in their downstream signaling pathways [11]. Current drugs with approved by the US Food and Drug Administration (FDA) were primarily VEGF antagonists (e.g., bevacizumab and sunitinib), multi-target TKIs (targeting VEGFR-3, including sunitinib and regorafenib) and VEGFR inhibitors such as cetuximab, erlotinib, and gefitinib. TKIs mainly inhibited angiogenesis, which was required for cancer cell survival and proliferation. All-grade and high-grade proteinuria reported in 33 clinical studies using VEGFR and TKIs were found in 18.7 and 2.4% of the individuals respectively [12]. Regorafenib was tolerated by Japanese individuals (*n* = 100) in the CORRECT (patients with metastatic colorectal cancer treated with regorafenib or placebo after failure of standard therapy) trial at a level generally comparable to other Asians; however, Japanese population showed elevated incidence rates of treatment-associated all-grade hypertension (60 vs. 23%), proteinuria (40 vs. 2%) and thrombocytopenia (39 vs. 9%), and elevated lipase amounts (25 vs. 2%) in comparison with non-Japanese cases [9].

Agents targeting VEGF attracted increasing attention in the treatment of late-stage cancer. VEGF was also a key molecule in the glomerular filtration barrier [13]. Normally, glomerular epithelial cells (podocytes) expressed VEGF, which acted on endothelial cells to maintain the structure and function of the glomerular filtration barrier. Animal experiments have shown that blocking VEGF could lead to endothelial cell damage, coagulation pathway activation, glomerular filtration barrier damaging, proteinuria promotion and TMA occurrence [12]. Glomerular microangiopathy caused by anti-VEGF treatment typically manifested as segmental glomerular capillary microaneurysms and hyalinosis, rarely with concomitant fibrin/platelet thrombi or broken red blood cells. Such microangiopathy was characterized by diffuse glomerular distribution, sparing pre-glomerular vessels, and morphological features of chronic TMA.

In recent years, the rapid development of new anti-tumor biological agents, such as ones with therapeutic inhibition of VEGF signaling, has brought about improved medical efficacy and provided patients with more choices, which however might also affect the functions of multiple organs, including the kidney. Many nephrologists were unfamiliar with kidney damage caused by these drugs, and oncologists had a hard time with it as well. With the necessity of novel anti-cancer drugs and increasing morbidity of renal problems associated with these agents, the need of establishing cross-discipline medical team was urgent, and nephrologists and oncologists should be encouraged to learn from and cooperate with each other in order to treat patients better. Specifically, the renal toxicity of anti-tumor biological agents should be listed as a discussion topic. At the same time, nephrologists should understand the prevention, clinical manifestations and management of cancerous lesions. While using bevacizumab, routine urine, blood pressure, liver and kidney function tests should be performed regularly. A reduction in immune tolerance and immune escape should be recorded as well, and the drug amounts should be reduced, in order to decrease the risk of albuminuria, nephrotic syndrome or renal insufficiency. However, the optimal way to monitor such patients still remained unknown. How susceptibility to VEGF suppression is regulated remains largely undefined. In addition, that an individual tolerating long-term treatment with bevacizumab develops TMA readily upon switching to regorafenib is enigmatic

In conclusion, this case indicates that regorafenib, a kind of TKIs may result in TMA, which is a rare but life-threatening complication of cancer treatment drug. Insights from this case might help physicians diagnose rare forms of TMA and adjust treatment for patients in a timely manner.

## Data Availability

The datasets used in this study are available from the corresponding author on reasonable request.

## Abbreviations

TKIs: Tyrosine kinase inhibitors
TMA: Thrombotic microangiopathy
RTKs: Receptor tyrosine kinases
VEGF: Vascular endothelial growth factor
VEGFR: Vascular endothelial growth factor receptors
mCRC: Metastatic colorectal cancer
PLA2: Phospholipase A2
THSD7A: Thrombospondin type-1domain-containing 7A
FDA: Food and Drug Administration
